# From clinical to molecular diagnosis: relevance of diagnostic strategy in two cases of branchio-oto-renal syndrome – case report

**DOI:** 10.1186/s13052-022-01369-5

**Published:** 2022-10-01

**Authors:** Elena Cacciatori, Sebastiano Aleo, Giulietta Scuvera, Chiara Rigon, Paola Giovanna Marchisio, Matteo Cassina, Donatella Milani

**Affiliations:** 1 Fondazione IRCCS Ca’ Granda Ospedale Maggiore Policlinico, Milan, Italy; 2 Università degli Studi di Milano, Milan, Italy; 3 Clinical Genetics Unit, Department of Women’s and Children’s Health, University of Padova, Via Giustiniani, 3, 35128 Padova, Italy; 4 Department of Pathophysiology and Transplantation, University of Milan, Milan, Italy

**Keywords:** Branchio-oto-renal syndrome, Deafness, *EYA1*, Copy number variation – case report

## Abstract

**Background:**

Branchio-oto-renal syndrome (BOR) is an autosomal dominant disorder characterized by deafness, branchiogenic malformations and renal abnormalities. Pathogenic variants in *EYA1*, *SIX1* and *SIX5* genes cause almost half of cases; copy number variants (CNV) and complex genomic rearrangements have been revealed in about 20% of patients, but they are not routinely and commonly included in the diagnostic work-up.

**Case presentation:**

We report two unrelated patients with BOR syndrome clinical features, negative sequencing for BOR genes and the identification of a 2.65 Mb 8q13.2–13.3 microdeletion.

**Conclusions:**

We highlight the value of CNV analyses in high level of suspicion for BOR syndrome but negative sequencing for BOR genes and we propose an innovative diagnostic flow-chart to increase current detection rate. Our report confirms a mechanism of non-allelic homologous recombination as causing this recurrent 8q13.2–13.3 microdeletion. Moreover, considering the role of *PRDM14* and *NCOA2* genes, both involved in regulation of fertility and deleted in our patients, we suggest the necessity of a longer follow-up to monitor fertility issues or additional clinical findings.

## Background

Branchio-oto-renal syndrome (BOR, OMIM # 113650) is an autosomal dominant disorder characterized by deafness, branchial cleft fistulae and cysts, malformations of the outer, middle, or inner ear, preauricular pits or tags, and a wide spectrum of renal abnormalities. BOR syndrome has an estimated prevalence of 1:40000 and contributes to approximately 2% of severe deafness in children [1]. Phenotypic features vary greatly among and within families; however, a near-constant clinical feature is hearing impairment (> 90%) [2]. This can present itself as conductive, sensorineural, or mixed, and can be more or less progressive [3]. The syndrome shares many features with the branchio-otic syndrome (BOS, OMIM # 602588) and therefore both conditions are considered as part of the branchio-oto-renal spectrum disorder (BORSD). Renal abnormalities are the only clinical feature that helps distinguish the two conditions. Major diagnostic criteria for BOR syndrome include second branchial arch anomalies, deafness, preauricular pits, auricular malformations, and renal anomalies; minor diagnostic criteria are external auditory canal, middle ear and inner ear anomalies, preauricular tags, facial asymmetry, and palatal abnormalities (see Table 1) [4]. A clinical diagnosis of BOR syndrome is established when an affected family member and a single major criterion are present. If family history is negative, three major criteria or two major and two minor criteria are required. This condition is mainly determined by disruptions in *EYA1* (OMIM * 601653, localized on chromosomal region 8q13.3), the human homologue of the Drosophila eyes absent gene, which encodes a transcriptional regulator essential for embryogenesis [5]. Approximately 150 different heterozygous pathogenic variants have been identified including frameshift, nonsense, missense and splice-site mutations (a full list of the variants can be found at: http://deafnessvariationdatabase.com/classification) [6]. These are responsible for about 40% of clinical BOR syndrome [4, 7], whereas approximately 4 and 5% of patients have pathogenic variants respectively in *SIX1* gene [8] and *SIX5* gene [9]. With more than 50% of patients remaining, the current diagnostic rate of BOR syndrome is unsatisfactory. It has been estimated that ~ 20% of individuals with BOR/BOS syndromes may harbor large copy number variants (CNV) and complex genomic rearrangements [4], as suggested by the description of patients with a dir ins(8)(q24.11;q13.3;q21.13) [10, 11], with a recurrent 8q13.2q13.3 deletion [12, 13], and with few non-recurrent deletions involving *EYA1* [14, 15]. Nevertheless, bibliographic references for genomic rearrangements are limited and old. Thus, a review of available data is necessary to improve diagnostic strategies and help recognize BOR patients.

**Table 1:** Diagnostic criteria for BOR syndrome*

Major criteria	Minor criteria
Branchial anomalies	External ear anomalies
Deafness	Middle ear anomalies
Preauricular pits	Inner ear anomalies
Renal anomalies	Preauricular tags
	Other: facial asymmetry, palate abnormalities

For a clinical diagnosis of BORSD, an affected individual must have at least three major criteria; two major criteria and at least two minor criteria; or one major criterion and an affected first-degree relative meeting criteria for BOR syndrome

*These diagnostic criteria were devised and published by Chang et al. (2004) [4]

Here, we describe two additional unrelated children carrying the recurrent 8q13.2q13.3 deletion and present a literature review on the clinical and genetic issues of the diagnostic workup in BOR syndrome.

## Case presentation

### Patient 1

The male proband is the second-born of non-consanguineous parents; family history was negative for genetic disorders. He was born at 40 weeks’ gestation after an uneventful pregnancy. His Apgar score at 1 and 5 min was 9 and 10, respectively, and auxological parameters were normal (birth weight 3250 g, length 49 cm, OFC 35 cm). At birth, bilateral external ear hypoplasia was noted. Audiological screening with otoacoustic emissions (OAE) and brainstem auditory evoked response (BAER) were normal; abdominal ultrasound showed the presence of mild left pyelectasis (anteroposterior diameter 5 mm). At the age of 5 months, a genetic evaluation showed the presence of single palmar creases, an auricular pit at the top of the right helix, and bilateral cervical fistulae. Cervical ultrasound showed the presence of two branchial cysts, while audiological evaluation did not reveal deafness; subsequently BAER revealed only a mild bilateral conductive hearing loss at the age of 2 years. Neurodevelopmental skills were normal. The presence of branchial cysts, cervical fistulae, external ear anomalies and mild renal pyelectasis suggested the clinical diagnosis of BOR syndrome and the analyses of *EYA1*, *SIX1,* and *SIX5* genes were performed by next generation sequencing (NGS). No abnormality was revealed. However, a read depth based, bioinformatic analysis of NGS data suggested that the proband carried a whole gene deletion of one copy of *EYA1*. Array-CGH confirmed the Copy Number Variant encompassing *EYA1*, detecting a de novo 2,65 Mb microdeletion located at 8q13.2–13.3 (chr8: 69,836,843-72,595,791 in genome build hg19).

### Patient 2

The male proband is the third son of non-consanguineous parents. Family history was negative. The child was born at 40 weeks’ gestation by vaginal delivery. Pregnancy was uneventful except for ultrasound detection of antenatal bilateral hydronephrosis. His Apgar scores at 1 and 5 min were 7 and 8, respectively, and auxological parameters were normal (birth weight 3450 g, length 50 cm, OFC 34 cm). Hypotonia and bilateral renal hypoplasia, with a resulting impaired renal function, were quickly revealed. During neonatal age, clinical and biochemical evaluation, along with ultrasound studies, showed the presence of bilateral cervical and preauricular fistulae, compatible with second branchial arch cysts. At 3 months of age audiological evaluation detected bilateral conductive hearing loss. Genetic evaluation showed the presence of bilateral preauricular pits. *EYA1*, *SIX1,* and *SIX5* gene sequence analysis by NGS, performed for the presence of four major criteria, returned negative but the CNV bioinformatic analysis suggested, again, a whole gene deletion of one copy of *EYA1.* An array-CGH was therefore performed and resulted in the detection of a de novo 2,65 Mb microdeletion located at 8q13.2–13.3 (chr8: 69,836,843-72,595,791 in genome build hg19).

## Discussion and conclusions

The subtle complexity of some genetic disorders is forcing clinicians to rely on multiple genetic tests to confirm a clinical diagnosis. By setting up a well-thought diagnostic strategy, an accurate diagnosis can be established in reasonable time and with minimal costs. In BOR syndrome the diagnostic workup usually starts with direct sequencing of *EYA1, SIX1,* and *SIX5* genes or a multigene panel which includes these same genes. This approach allows the genetic confirmation of almost half the cases and reduces the likelihood of detecting variants of uncertain significance (VUS). Here, we report the diagnostic workup used in two unrelated patients whose clinical features included deafness, auricular anomalies, auricular pits/tags, renal and second branchial arch anomalies, thus fulfilling the clinical criteria for BOR syndrome. *EYA1, SIX1* and *SIX5* gene sequence analysis were performed but returned negative in both patients. A copy number variation (CNV) analysis was subsequently carried out and led to the identification of a 2.65 Mb microdeletion located at 8q13.2–13.3 (chr8: 69,836,843-72,595,791 in genome build hg19) encompassing eight genes (*SULF1, SLCO5A1, PRDM14, NCOA2, TRAM1, LACTB2, XKR9, EYA1*). Microdeletions including *EYA1* have been previously reported in a limited number of patients: the first cases date back to 2004, when some of the more sophisticated diagnostic tests to detect CNVs were not habitually employed; however, with the implementation of array-CGH and MLPA analyses in clinical practice, an increasing number of CNVs have been reported in the literature and in the available databases [12]. Therefore, our two cases confirm the importance of CNV analysis in the diagnostic process of patients with no mutations detected by BOR genes sequencing.

NGS platforms are now available in most medical genetics laboratories and allow, with a single test, both the sequencing and the CNV analysis of the three main BOR-associated genes; CNV calling is carried out using specific bioinformatic tools, which usually reliably detect multi-exon CNVs but show poor performance in case of small CNVs involving only one or a few small exons. Currently, this bioinformatic analysis is not considered the gold standard for CNV detection and any results need to be confirmed by MLPA; however, it is a useful and quick screening tool which does not require additional costs other than those related to sequencing. Therefore, MLPA analysis should be performed in all cases with a negative sequencing test, regardless of the CNV detection at the bioinformatic analysis. However, when a whole gene involvement is observed, even if only by bioinformatic analysis, the inclusion of other genes should be investigated by an array-CGH analysis with an adequate resolution. The latter could be employed as a first test in patients displaying complex and unusual clinical features, such as severe intellectual disability. Furthermore, when evident clinical signs of BOR syndrome are present, a conventional cytogenetic test may also be taken into account if both NGS and CNV analyses are negative: although rare, balanced inversions and translocations have been described and may go undetected when using array-CGH or MLPA [16, 17].

**Fig. 1:**
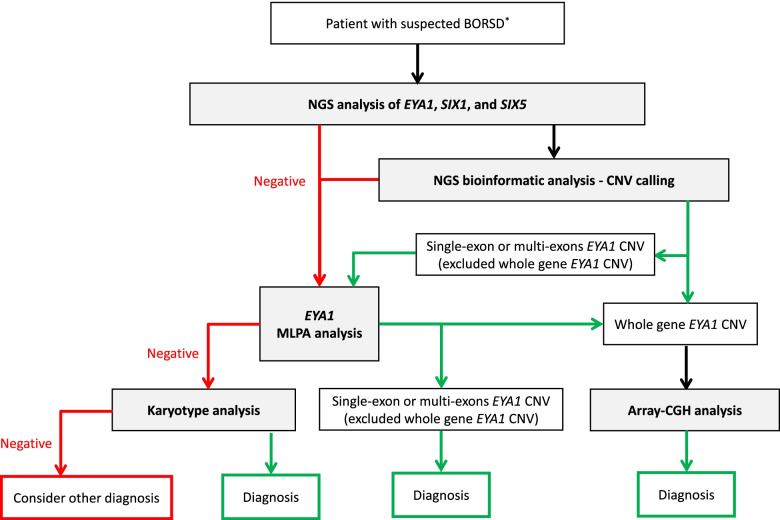
Diagnostic flowchart for BORSD. Green arrow: positive test; red arrow: negative test. CNV: Copy Number Variant. *See Table 1 for the Diagnostic Criteria of BOR syndrome by Chang et al. (2004) [4]

Based on our suggestions, we propose a diagnostic flow chart as an instrument to increase the current detection rate of BORSD (see Fig. 1).

Both the patients described in our report show the same 2,65 Mb microdeletion located at 8q13.2–13.3. Reports describing 8q13.2–13.3 microdeletion carriers with similar breakpoints are scarce. In one paper, Sanchez-Valle et al. proposed that human endogenous retroviral (HERV) blocks (remnants of ancient retroviral infections which can cause genomic rearrangements) may have been involved in one of their patient’s 2.7 Mb microdeletion, and that contiguous gene deletion involving *EYA1* may contribute to developmental delay, particular musculoskeletal features, and some classic signs of BOR syndrome [14]. Similar breakpoints were later found in four BOR patients by Brophy et al. [15] and by Chen et al. [18], who explained the recurrence with a mechanism of non-allelic homologous recombination (NAHR), mediated by HERV sequences. The HERV hypothesis was also maintained by Men et al., who reported three BOR patients from the same family with a 2,69 Mb deletion at 8q13.2–13.3 involving the whole *EYA1* gene and breakpoints residing in the LTR elements of HERV sequences [19]. Our patients seem to confirm that a recurrent pathogenic mechanism underlies this kind of microdeletion. Sometimes, however, BOR syndrome may also be caused by atypical microdeletions, which are thought to arise from different mechanisms from those just described. Patients with atypical microdeletion can show clinical features not included in the typical criteria for BORSD. Sanchez-Valle et al. described two BOR patients harboring a 3.6 Mb and an 8.7 Mb deletion both including *EYA1*, and a clinical phenotype characterized by variable intellectual disability, short stature, dysmorphic features, growth hormone deficiency, and hypotonia. The same authors suggest that these additional features may be explained by a greater extension of the atypical microdeletions [14]. In contrast, our two recurrent 8q13.2–13.3 microdeletions show similar clinical features to those seen in individuals with intragenic *EYA1* variants suggesting that the available criteria for BOR diagnosis are also valid for patients with the recurrent microdeletion involving *EYA1*. It should be noted, however, that atypical features have been reported in some patients with BOR syndrome caused by the recurrent microdeletion. These include microphthalmia with iris and retinal coloboma and clubfoot [14]. Au et al. also described the presence of arthrogryposis in a patient with an otherwise clinical phenotype of typical BOR syndrome; they speculated that this characteristic feature, previously undescribed in patients with this microdeletion, may be related to the haploinsufficiency of the *NCOA2* gene (OMIM * 601993), encoding the steroid receptor coactivator 2, involved in muscular differentiation [12]. Arthrogryposis, however, was not noted in our two cases nor in other described BOR patients with deletions involving *NCOA2* [14, 15, 18, 20]. This suggests that the deletion of *NCOA2* may not be sufficient for arthrogryposis to develop.

It has now been 3 years since our two patients were diagnosed. To exclude the potential onset of additional atypical findings, a longer follow-up period is probably necessary. Considering that approximately 90% of BOR patients with a point mutation have an affected parent [21] and that most 8q13 microdeletions seem to be de novo, it is possible that the latter is associated with a reduced genetic fitness. Among the genes found to be deleted in our patients, *PRDM14* (OMIM * 611781) and *NCOA2* (OMIM * 601993) have been shown to play an important role in fertility. Studies in mice provided evidence that Prdm14 is involved in specification of primordial germ cells; specifically, the data suggest it has an essential role in the reacquisition of potential pluripotency of germ cells and their subsequent genome-wide epigenetic reprogramming [22]. Studies with *NCOA2*-null (−/−) mice demonstrated fertility impairment of both sexes; male hypofertility was due to teratozoospermia and age-dependent testicular degeneration, while female hypofertility was due to placental hypoplasia [23]. In addition, the testicular Androgen Receptor activity was decreased significantly in *NCOA2* +/− mice [24].

Interstitial deletions at 8q13 have been identified in patients affected by mesomelia–synostoses syndrome (MSS) (OMIM # 600383), an autosomal dominant disorder characterized by mesomelic limb shortening, acral synostoses, and multiple congenital malformations; the deletions varied in size, but all encompassed only *SULF1* and *SLCO5A1* genes [25]. These genes are both included in the recurrent 8q13.2–13.3 microdeletion but none of the patients displayed the typical features of MSS; therefore, haploinsufficiency of these two genes cannot be considered the pathogenic mechanism underlying this disorder. It has been proposed that MSS is the result of the disruption of a topological associated domain (TAD) boundary within *SULF1* [26].

BOR syndrome is characterized by an extreme intra- and interfamilial clinical variability; the two patients we have described carry the same genetic alteration and confirm the variable expressivity of the associated phenotype. However, to date no precise genotype-phenotype correlations have been defined for BOR syndrome, even for point mutations [27].

In conclusion, this study describes the clinical features of two unrelated patients affected by BOR syndrome due to a recurrent 8q13.2–13.3 microdeletion and underlines the unsatisfactory diagnostic rate in these patients. Analyzing our cases’ management, we go over the main laboratory techniques that are used to diagnose BORSD and develop a diagnostic flow chart which aims at improving the detection rate of this condition. We also highlight the increasing evidence of a recurrent mechanism possibly responsible of the typical 8q13.2–13.3 microdeletion found in our patients and consider that a longer follow-up period might be necessary for revealing additional clinical findings.

## Data Availability

The data generated during the current study are not publicly available due to privacy or ethical restrictions.

## Abbreviations

BOR: Branchio-oto-renal syndrome
CNV: Copy number variants
BOS: Branchio-otic syndrome
BORSD: Branchio-oto-renal spectrum disorder
OAE: Otoacoustic emissions
BAER: Brainstem auditory evoked response
NGS: Next generation sequencing
Array-CGH: Array -Comparative Genomic Hybridization
VUS: Variants of uncertain significance
MLPA: Multiplex Ligation-dependent Probe Amplification
HERV: Human endogenous retroviral
NAHR: Non-allelic homologous recombination
LTR: Long Terminal Repeat
MSS: Mesomelia–synostoses syndrome
TAD: Topological associated domain
